# Reward, Context, and Human Behaviour

**DOI:** 10.1100/tsw.2007.122

**Published:** 2007-05-29

**Authors:** Clare L. Blaukopf, Gregory J. DiGirolamo

**Affiliations:** Department of Experimental Psychology, University of Cambridge, UK

**Keywords:** reward, punishment, context, saccades, brain systems, consciousness

## Abstract

Animal models of reward processing have revealed an extensive network of brain areas that process different aspects of reward, from expectation and prediction to calculation of relative value. These results have been confirmed and extended in human neuroimaging to encompass secondary rewards more unique to humans, such as money. The majority of the extant literature covers the brain areas associated with rewards whilst neglecting analysis of the actual behaviours that these rewards generate. This review strives to redress this imbalance by illustrating the importance of looking at the behavioural outcome of rewards and the context in which they are produced. Following a brief review of the literature of reward-related activity in the brain, we examine the effect of reward context on actions. These studies reveal how the presence of reward vs. reward *and* punishment, or being conscious vs. unconscious of reward-related actions, differentially influence behaviour. The latter finding is of particular importance given the extent to which animal models are used in understanding the reward systems of the human mind. It is clear that further studies are needed to learn about the human reaction to reward in its entirety, including any distinctions between conscious and unconscious behaviours. We propose that studies of reward entail a measure of the animal's (human or nonhuman) knowledge of the reward and knowledge of its own behavioural outcome to achieve that reward.

## WHAT ARE REWARDS?

Rewards are a fundamental influence in the behaviour of animals. Humans and other animals are driven to seek out and attain rewards and to avoid punishments or penalties. Rewards are classified into two types. Primary rewards do not need to be learnt and they serve basic needs for survival and reproduction. They include food, water, and sex. Secondary rewards are more abstract and cognitive in nature, and their reward value must be learnt. Examples are money, acclaim, security, knowledge, and praise. Some secondary rewards are more closely related to basic needs and survival than are others. For example, money can buy food and shelter, and security improves your chances of survival. On the other hand, achieving a particular body shape because your culture deems it attractive is less straightforward. If the secondary reward (achieving a particular body shape) has been assigned a positive value in our culture, then it may increase the chances of finding a mate and, hence, act as a positive reward. However, potentially maladaptive behaviours (eating disorders, stress, greed) are also produced by these secondary rewards. Understanding secondary rewards is critical to understanding human behaviour in our society.

Rewards have several basic functions. Intuitively speaking, a reward is something one receives for completing something or doing well in a task (for example, rewarding yourself with a large slice of cake after having just completed a 10-mile run). In addition, rewards produce feelings of pleasure and liking, and reinforce the behaviour realising them — you are more likely to go on a long run again when you think about the large slice of cake you enjoyed so much last time. The psychological components of reward can be decomposed into three critical components[1,2]: (1) learning about relationships among stimuli and about the consequences of actions, including implicit and explicit knowledge produced by associative conditioning and cognitive processes; (2) affective or emotional components — implicit "liking" and conscious pleasure produced by reward consumption; and (3) motivated behaviour — implicit incentive salience "wanting" and cognitive incentive goals, i.e., instrumental performance and consumption of rewards.

To further our understanding of the psychological components of reward, particularly as they relate to secondary rewards so critical and unique to the human animal, it is important to reveal how reward influences behaviour. Each of the three components of reward can be characterised as having a conscious or unconscious element. The conscious aspects of reward are widely acknowledged to be difficult to study in animals, such as the emotional component of rewards (e.g., "pleasure"). This review will concentrate on the third component, motivated behaviour, and will illustrate the value in studying human subjects, particularly through the examination of distinctions in conscious and unconscious behaviour.

## HOW ARE REWARDS PROCESSED?

This section outlines the critical brain areas and mechanisms in the reward system of the brain. Evidence will be provided both from humans and from animal models. Most of our knowledge of reward-related brain activity is from animal studies, which use methods such as single-cell recording, brain lesions, psychopharmacology, electrical self-stimulation, and administration of addictive drugs. However, limitations exist in using animal studies to build a complete understanding of the processing, functions, and effect of rewards in humans. In particular, animal studies usually only employ basic rewards because cognitive rewards are difficult to investigate in the laboratory animal. Recently, the development and improvement of imaging techniques has led to an explosion of reward-related studies in humans. Functional magnetic resonance imaging (fMRI) is currently the most useful tool for investigating the reward systems in the human brain, although event-related potential (ERP) recordings and occasionally, single-cell studies have been revealing.

Though the regions activated depend on the task used, there is a consistent set of reward-related neural structures found in humans. It is clear that the regions identified in the human neuroimaging work parallel those identified in the extensive animal literature, even when bearing in mind that the spatial resolution of fMRI is not as high as that of single-cell studies. Abstract, secondary rewards used in the studies of humans are associated with neuronal responses in the same regions that respond to primary rewards in animals. This kind of common network, for both secondary and primary rewards, would allow very different rewards to be compared directly to each other, in order to choose between possible courses of action[3].

Many studies have focussed on the detection, prediction, and valuation of rewards. The set of brain areas consistently activated in this work includes the striatum (caudate nucleus, putamen, and ventral striatum, including the nucleus accumbens), the amygdala, dopaminergic neural areas in the midbrain, and orbitofrontal cortex[4,5]. The less well-studied aspect of reward processing is the integration of reward information for the purpose of action. The set of brain areas implicated in the production and modulation of motivated behaviour includes the anterior cingulate cortex, the basal ganglia, (dorso)lateral prefrontal cortex, parietal cortex, superior colliculus, and premotor cortex. Various studies have attempted to attribute distinct reward-related functions to each of these areas and some of these are described in the subsections below. It should be noted, however, that neural areas often appear to have more than one role and are active in multiple situations, so this summary defines general properties, but is not complete or restricted. Indeed Roesch and Olson[6] underline the need for more work in this area when illustrating how difficult it is to disambiguate the functional significance of modulated neuronal activity recorded in nonhuman primates. Activity may represent the value of an expected reward or activity may reflect motivational modulation of motor signals.

## Reward Detection, Prediction, and Expectation

Much of our current knowledge about the detection, prediction, and expectation of rewards comes from electrophysiological studies of single cells in nonhuman primates. Results are reviewed more fully elsewhere[7], so no more than a brief summary is presented here before addressing the human functional anatomy.

There are a large number of brain areas that respond to the presence or delivery of rewards. This set of brain areas includes cortical areas, such as dorsolateral prefrontal cortex, orbitofrontal cortex, anterior cingulate cortex; and subcortical areas, such as the striatum (caudate nucleus, putamen, and ventral striatum, including nucleus accumbens), subthalamic nucleus, pars reticulata of the substantia nigra, the lateral hypothalamus, and the amygdala. In addition, the striatum, amygdala, and orbitofrontal cortex contain neurons that respond when a reward is expected, but has not yet been presented. For example, several studies have demonstrated that cell responses in the caudate reflect both the target of an upcoming saccade and the reward expected after making the movement[8,9].

The response of mesencephalic dopamine neurons to the delivery of reward has attracted particular interest. A role for dopamine in the neuronal mechanisms of reward was first implicated many years ago[10]. For a long time, it was assumed that dopamine was responsible for the hedonic feeling (pleasure) associated with receiving rewards. However, dopamine is not necessary or sufficient for generating “liking” for sweet rewards in animals, and accumulating evidence suggests that it does not mediate subjective pleasure of drug rewards in humans. Currently, there are many suggestions for the role of dopamine, including the “wanting” (or motivational) component of reward, the signalling of reward prediction errors, and others[11,12]. Dopamine acts at several different timescales and this may reflect several complementary functions that are dependent on the rate at which its concentration fluctuates[13]. For example, dopamine exerts a tonic influence via its continuous, low extracellular concentration in dopamine-innervated areas and this is crucial for enabling a large number of behavioural processes, such as movement, cognition, and motivation. Other factors cause a slower modification of the central dopamine level over seconds and minutes, and may mediate the processing of reward, feeding, drinking, punishment, stress, and social behaviour[14]. However, the most relevant aspect of dopamine firing for the present discussion is the phasic response to the delivery of rewards and to stimuli that predict rewards, with a time course of only tens of milliseconds[15,16]. This phasic response may not actually code reward itself, but rather a reward prediction error[17]. This idea has received much support from recordings in nonhuman primates[18]. Importantly, phasic response is elevated following surprising rewards, depressed following omitted rewards, and is unaffected by correctly predicted rewards[13].

In humans, dopaminergic neurons are more difficult to study. However, they project to other areas that can be examined relatively easily using functional imaging techniques, including the striatum and most areas of the neocortex, with particular focus on the prefrontal cortex. In humans, activity is consistent with reward prediction errors for appetitive events in the ventral striatum[19] and also the orbitofrontal cortex[20], and for aversive events in the ventral striatum[21]. Jensen and colleagues[22] found that the ventral striatum activations are consistent with signalling an error in predicting the salience of a stimulus regardless of its valence, with both appetitive and aversive events being equally effective. Ventral striatum activity is greater when the rewarding stimuli are unpredictable[23] and, importantly, increases with anticipation of increasing monetary rewards[24]. This latter result suggests an important role for the striatum in secondary rewards, and underlines how the same area is active in both primary and secondary rewards.

Although the discussion so far has focussed on the impact of reward in the ventral striatum, activity in the dorsal striatum is also strongly influenced by reward. In nonhuman primates, neurons in the caudate and putamen show reward-expectation activity[25]. In addition, many behaviour-related activations in the dorsal striatum, such as expectation and detection of instruction or stimuli, and the preparation, initiation, and execution of movement, show relationships to reward[26]. Human neuroimaging studies have found dorsal striatum activity that is affected by both magnitude and valence of monetary outcomes[27]. Therefore, reward-related processes happen throughout the striatum. However, the ventral and dorsal parts of the striatum have different roles[6]: the ventral striatum is limbic in nature (the amygdala projects mainly to ventral parts of the striatum); the dorsal striatum is one of the structures that appears to be interposed between limbic and motor systems (all subdivisions of prefrontal cortex, premotor cortex, and primary motor cortex project to different parts of the dorsal striatum). The dorsal striatum may therefore relay information between evaluative responses to reward and the actions related to these rewards. The anatomical relationship between motor and reward-sensitive neural areas highlights the importance of reward on actions.

Thus, it is not surprising that reward detection, prediction, and expectation encoding is also found in cortical premotor areas. For example, reward-predicting and reward-detecting neurons have been found in the supplementary eye fields[28]. The reward-predicting neurons linearly increase their firing shortly before saccade onset and continue until the reward presentation, long after the initiation of the saccade (300–500 ms after). In contrast, reward-detecting neurons fire in phase with reward delivery. In addition to this activity in the supplementary eye fields, reward expectancy is also represented in the activity of supplementary motor area neurons, even in the context of an oculomotor task[29]. These data, taken together, suggest a reward-expectancy signal that is present throughout the dorsomedial frontal cortex.

## Reward Value and Preference

Current stimulus-value associations require the acquisition and rapid updating of representations of reinforcer value, and the linking of this potentially changing value to the stimulus. Rapidly updated stimulus-value associations that support instrumental behaviour and goal-directed action are mediated by the basolateral complex of the amygdala[30]. Neurons in this area that fire selectively in response to food can be modulated by salting the food, presumably changing its affective significance[31,32].

The amygdala is well known for its association with fear and negative emotions. However, it has now also been consistently found that amygdala neurons respond to positive stimuli[33] and rewards[34], and in particular seem to encode the intensity or magnitude of rewards[35,36]. Reward-related neurons in the basolateral amygdala of rats anticipate reward encounter, respond during reward consumption, and differentiate between high and low reward magnitude[37]. Via the use of olfactory and gustatory stimuli matched for valence and differing in intensity, the amygdala has also been implicated in processing reward intensity in humans[35,36]. In this study, the orbital frontal cortex (OFC) was implicated in coding for valence, whereas the amygdala was exclusively involved in processing odour intensity. Therefore, it seems that the amygdala is encoding how arousing stimuli are. Previous theories associating amygdala activity with only negative stimuli may simply reflect the stronger salience of negative events. The value of a stimulus is clearly an interaction of valence and intensity.

The OFC is crucial in the process of reward valuation[38]. Clearly, neurons in the OFC are also able to distinguish between rewards and punishers[39]. The OFC encodes stimulus-reward value from different sensory modalities[40] as well as more abstract, secondary rewards, such as money[41]. In addition, the OFC tracks the changing values of rewards. For example, the response of neurons in monkey OFC to a particular taste varies according to whether the animal has been fed to satiety with that food or is hungry[40]. Similarly, if hungry human participants are scanned in the presence of two food-related stimuli and then fed to satiety on one of these, OFC activity to the eaten food decreases, but activity to the food not eaten remains the same[42]. Studies of humans with OFC lesions show how important this region is in altering and guiding voluntary action based on such changing reward values and contingencies[43,44].

The OFC is particularly important in the relative coding of rewards. Reward discriminations are based on the relative preference for the available rewards reflected in the animals' choice behaviour, rather than any physical or other absolute reward properties. Thus, neurons in the OFC appear to process the motivational value of rewarding outcomes and may be critical for voluntary action[45]. This encoding of relative motivational values to action outcomes might be important input for neuronal mechanisms in the frontal lobe that underlie goal-directed behavioural choices[46]. Relative coding means that the neuronal response to a reward varies according to other rewards available at that time. In short, reward-related neural activity depends on the context in which the reward is presented. This is a critical idea to both this review and the literature in general. Hence, a particular reward could be the preferred option in one context, but the nonpreferred option in another.

There may be functional heterogeneity within the OFC, with a role for subregions in representing stimulus-reward values, signalling changes in reinforcement contingencies and in behavioural control[47]. The human OFC is also sensitive to the context in which a reward is presented. The presence of punishments and rewards together produce different activity in the OFC compared to rewards alone[41,48]. This contextual activity may also be reflected in different regions within the OFC. Both medial and lateral regions of the OFC show an enhanced response to the lowest and highest reward values relative to the midrange[41,49]. However, in a different context (the presence of both reward and punishment), medial OFC response correlates with reward value, while lateral OFC correlates with punishment value[48]. The prospect of negative outcomes may lead to functional dissociation between medial and lateral regions. Other studies have found similar results[35,38,50,51]. In addition to lateral OFC, other studies have highlighted the importance of the anterior insular cortex in representing aversive value[22,52,53,54]. Because lateral OFC responses are particularly associated with behavioural inhibition and perceptual set shifting[55], punishment may more efficiently initiate a behavioural change. As we shall see, the prospect of punishment changes the behavioural consequences of reward in humans[56].

## Reward to Motor Output

The anterior cingulate cortex (ACC) appears to have a crucial role in the control of reward-guided behaviours, relating actions to their expected consequences and guiding decisions. Neurons in the cingulate motor area may be important for the modification of behaviour towards a reward when the motivational value changes. In monkeys, these neurons are selectively active when switching to a different movement because producing the current movement would lead to less reward[57], which suggests a role in selecting movements for maximum reward. Anatomical studies have revealed that the cingulate motor area is in a key position to process the information necessary to select voluntary actions in accordance with the subject's internal and external requirements[58]. Anatomically, the ACC receives information from the limbic structures and the prefrontal cortex about motivation and the internal state of subjects, as well as a cognitive evaluation of the environment. In addition, the ACC output goes to primary and secondary motor areas, and other motor structures in the brainstem and spinal cord. ACC activity correlates to task value and the size of the rewards received, where an optimal stimulus (the stimulus with the highest probability of reward) must be identified[59]. Other areas that assist in the control of reward-guided behaviours are the dorsal striatum and the dorsolateral prefrontal cortex. Their neuronal activity signals both the nature of the planned action and the value of the expected reward[6].

In humans, activations in areas of the medial prefrontal cortex (PFC) and anterior cingulate gyrus have been observed in a number of studies of reward processing[60, 61]. Again, these regions appear to be involved in the complex process of integrating reward information in action and decision making[62]. A medial-frontal ERP, localized to the ACC, is found after subjects made a choice between two cards in a gambling task and it is larger for losses than for gains[63]. This activity is found within 265 ms of presentation of the choice and is consistent with rapid evaluation of the motivational impacts of events and subsequent guiding of behaviour. In accordance, affective responses are faster and stronger to negative events than positive ones[64]. Moreover, single-cell neural activity in dorsal ACC neurons increases when human subjects are instructed to change the direction of a joystick movement, and the greatest response occurs when the change in direction signifies a reduced reward[65]. After dorsal ACC ablation, subjects make more errors when required to change behaviour based on reward reduction, providing further evidence for the importance of dorsal ACC in relating reward-related information to the selection of alternative actions, especially in the context of diminished return[66]. The dorsal ACC shows greater responses when deciding between two objects to gain the lesser punishment, but the ventromedial PFC showed greater responses when deciding between two objects to gain a greater reward, showing a functional distinction between different areas within the medial frontal cortex[67]. This increased activation in the dorsal ACC may reflect increased response competition when choosing between negative rather than positive options. Reaction times and error rates are much higher for this type of decision. These data reflect the importance of punishment on response choice with specific modulation of the more motoric aspects of the action being modified by reward and punishment.

The dorsolateral PFC plays an important role in holding visuospatial information online to guide behaviour for all effectors, from movements of the hands to movements of the eyes. The lateral prefrontal cortex (LPFC) is a likely place where the integration of reward processing and spatiomotor processing occurs. This area receives the necessary projections from the OFC and midbrain dopamine areas (for reward processing) and is well characterized as holding information online to guide behaviour[68]. In accordance with this view, Kobayashi and colleagues[69] found several subsets of neurons in LPFC: S-type cells that coded the spatial location of a to-be-made saccade, R-type cells that coded the reward, and SR-type cells that coded both. The SR-type cells correlated with the modulation of saccade behaviour by expected reward outcome, providing strong evidence for the integration of reward and action within the LPFC. Additionally, performance was better and more precise in reward-present trials compared to reward-absent trials. The PFC may change the goal aspects of action based on expected reward outcome, and, as we shall see, the basal ganglia may change the motor aspects of action.

Modulation of neuronal activity by reward value (higher neural activity after cues predicting large rewards) is more prominent the more posterior the brain area, in both lateral and medial frontal areas[6]. Therefore, two distinct processes may happen in the brain when predicting a reward: the first process occurs in the OFC and involves representing the value of the reward; the second process is manifest in premotor cortex and involves maintaining a level of motivation consistent with the value of the reward[70].

## WHAT ROLE DOES CONTEXT PLAY?

Importantly, we have seen context modify the functional response of the human and nonhuman brain. As discussed before, different patterns of activation are found within the OFC, and this difference is best understood as a change in context. When only reward is presented, both medial and lateral regions of the OFC show a correlation with reward value[41,49]. In contrast, when reward and punishment are present, the medial OFC response correlates with reward and lateral OFC correlates with punishment[48].

Likewise, there is evidence that ACC activity is determined by the value of the outcome relative to the range of possible outcomes and not the absolute value of the outcome. When presented with three possible outcomes, where the objective value of the middle one is halfway between the objective values of the best and worst outcomes, the reward-prediction system will come to predict the middle value. The mesencephalic dopamine system conveys this reward prediction signal and the ACC uses it to improve performance on the task. Outcomes are then judged relative to this expectation (the middle value), and departures from this produce relative increases or decreases in cingulate activity[71]. Hence, ACC activity is context dependent.

Other areas within and outside of the traditional reward areas are also influenced by contextual information[72]. Reward-related activity in the basal ganglia, PFC, posterior cingulate, inferior parietal lobule, and cerebellum (with the exception of the right cerebellum and left medial frontal gyrus) show context-dependent activation based on the range of rewards. Bilateral anterior cingulate and medial PFCs are specifically activated when the context remains constant in a rapidly changing sequence of rewards or punishments[73]. However, a change in context (going from a reward to a punishment or vice versa) activated the right dorsolateral PFC. Moreover, the LPFC not only predicts the absence of reward, but also represents more specifically which kind of reward will be omitted in a given trial[74]. Therefore, these neurons seem to code contextual information and tonic baseline activity in the LPFC may be related to monitoring such motivational context. This kind of recent trial history context also modulates dopamine neurons[75]. The dopamine reward-prediction error is a context-dependent prediction error, using both the recent trials and overall experimental context.

Subjects' intentions also seem to modulate reward-related activity in cortical and subcortical neural areas. The caudate nucleus is robustly activated only when subjects think that their button press determines whether they won or lost money. The dorsal anterior cingulate is preferentially activated when subjects make an action based on their own motivation and OFC activation is negligible. The reverse is found when subjects are told what response to make[76]. These results show the motivational significance of an action on reward-related neural areas.

These studies illustrate how important it is to take into account the overall context of an experiment when drawing conclusions about reward-related brain activity and the effect of rewards on actions. The range of possible outcomes, including the presence or absence of possible punishment, the recent outcome history, and motivation of the action, are all factors likely to have an effect.

## WHAT IS THE EFFECT OF REWARD ON OUR ACTIONS?

The anticipation of reward leads to motivated behaviour. For this behaviour to be labelled goal-directed behaviour, the reward and the contingency between action and reward both need to be represented in the brain during the action[77]. As we have seen, the ability to represent the value of rewarding and punishing events, estimate when and where such rewards and punishments will occur, and use this information to modulate behaviour, are all at the heart of goal-directed behaviour. We use here the eye movement system as our model of behaviour that is influenced by reward or punishment. We use the eye movement system (i.e., the system responsible for selecting and executing saccades) because eye movements are a convenient and easily measured example of voluntary and reflexive behaviour (e.g., antisaccades and prosaccades, respectively), and the anatomy and neural connectivity of the eye movement system is particularly well known, allowing us later to discuss the neural mechanisms behind the effects that we describe here. It is now widely accepted that voluntary eye movements made by humans in natural situations are guided by internal reward[78] but very little is known about the quantitative effects of reward and punishment on human saccadic programming.

In general, the nonhuman primate results show that speed and accuracy of actions are enhanced when a more-valued reward is expected[69,79,80,81]. These studies focus on the behavioural effects of reward alone. As we have discussed above, the brain's response to rewards alone is different from the context of both reward and punishment. As we will see below, the effects of reward on human behaviour are labile, depending on whether punishment is also a possible consequence.

There are obviously important differences between humans and animals in potentially rewarding situations. With humans, you can explain the reward contingency verbally, and you can ask them to repeat it back to you to check if they have understood it. Humans are also very good at working towards future goals with no immediate rewards, and will continue to carry out the correct movements trial after trial without reinforcement. This is in addition to the difference in the types of reward used: primary rewards, such as food pellets, are used in animal studies and more complex secondary rewards, such as money, are used in studies with humans.

Jazbec and colleagues[82] found results in humans that mirror those in nonhuman primates, despite the differences in reward type. In trials where the reward, punishment, or neutral value of the current trial was signalled well in advance, subsequent eye movements in reward and punishment trials were faster than those in neutral trials, and performance was more accurate. Subjects were given feedback at the end of every trial, informing them of their performance and their monetary reward or punishment.

Mimicking real-life situations where reward is not defined ahead of time, but needs to be determined online, two recent studies[56,83] used an antisaccade task where the reward value of the current trial was signalled by the go signal itself. No feedback was given at the end of each trial. In this situation, both the eye movement programming and the interpretation of the reward information must be completed in the short period before the eye movement is initiated (~200 ms for errors, ~350 ms for correct actions). In addition, subjects were working on the promise of a future reward and were not reinforced after every action, again reflecting more realistic situations. The paradigm employed was a gap antisaccade task[84] where subjects make 15–20% errors. On most of these error trials, subjects corrected their errors extremely quickly. Remarkably, on an average of 50% of error trials, subjects do not realise they have made this marked departure from the single correct eye movement: They do not report that they have made an error, so the error and corrective action are unconscious. The results from the first of the two studies agreed with the animal results, in the sense that movements in the potentially more rewarding condition were faster, but only conscious errors were significantly affected. When correct antisaccades or unconscious errors were made, there was no significant effect of reward, but when conscious errors were made (subjects made prosaccades towards the go signal), they were significantly faster when the go signal signified a higher reward value. This speeding was found even though the subjects would not receive that higher reward as they had made an error. The reward potential influenced action even in the absence of the correct action that would gain the reward. These results may reflect the distinctions of rewards having an implicit and explicit component, with differential effects on behaviour based on the conscious or unconscious action.

In another study[56] reflecting the differential neural response to reward alone vs. reward and punishment, subjects were rewarded for a correct movement or punished for an incorrect movement, and the motivation levels were increased. Under these conditions, both reward and punishment induced a significant change in all types of movement made. The monetary value of the trial was able to affect the reaction time of antisaccades, conscious errors, and unconscious errors. The saccades in high-reward and high-punishment trials were slowed compared to saccades in low-reward and low-punishment trials, respectively. However, unconscious errors were differentially affected as they were speeded when punishment was high. Therefore, the effect of reward is not static. Movements in a rewarded condition are speeded compared to the nonrewarded condition[82]. However, much like the neural changes based on context, the behavioural response also changes if the context is different. If there is the possibility of punishment on some trials, and the trial value is not known well in advance, the influence of reward changes.

The results of these studies also show us that rewards can act on a very short timescale. The saccades that were initiated in less than 200 ms were differentially affected by the various monetary values[56]. Therefore, subjects were processing the colour of the go signal, associating the colour with the monetary value for that go signal stored in memory, and programming the saccade all within that short time.

The advantages of using saccades to investigate these phenomena are that saccades and visual processing are tightly linked, and that the saccadic neural circuitry is well understood. The visual information can very quickly access structures involved in the planning of the eye movement, and any change in reaction time brought about by these influences will be noticeable. With manual responses, actions are slow enough that preparation and conscious control can have qualifying influences. Therefore, it would not be surprising if a manual analogue of this task does not show the same effects of reward and punishment (and preliminary results suggest this is the case). This is a largely neglected area of study, but there are a handful of studies looking at the contextual effects of monetary rewards and punishments on other aspects of human behaviour besides eye movements.

Trommershauser and colleagues[85] investigated the effects of rewards and punishments on the planning of manual responses. They asked subjects to rapidly touch a computer screen displaying a reward region and an adjacent penalty region. If subjects touched the reward region, they received points

(later translated to monetary reward); if they touched the penalty region, they lost points. The two regions were overlapping by various amounts and if both regions were touched, then both the reward and penalty were received. The penalties were varied, but the rewards were fixed. There was also a large penalty for not touching the screen in time. The penalty region was displayed for 500 ms before the reward region appeared, and the reward region acted as the signal to start the movement. Therefore, the location of the reward region and its distance from the penalty region were not known before the start of the trial. Subjects shifted their mean points of contact with the computer screen in response to changes in penalties and the location of the penalty region relative to the target region. There was no change in reaction time in the different conditions. Thus, manual movement planning can take into account possible punishment, but this was manifested as a change in movement endpoint and not as a change in reaction time.

Rewards and punishments can also influence covert mental processes, such as visual attention. Using a negative priming paradigm with variable monetary rewards as arbitrary feedback on performance, one experiment[86] tested whether the lingering inhibition of distracters was stronger after highly rewarded responses than poorly rewarded responses. Negative priming was strong following highly rewarded selections and was eliminated after poorly rewarded selections. The efficacy of visual selective attention can be adjusted by reward and punishment.

As revealed above, a major advantage of studying humans is that all aspects of reward function can be investigated. For example, the distinction between conscious and unconscious components of actions can be examined[56], as can the affective or emotional aspects. Berridge and Winkielman[87] have demonstrated a distinction between conscious and unconscious “liking” in humans. They found that a subliminally brief view of a happy facial expression produced no change in subjective feeling or mood ratings at the moment it occurred, but it still caused thirsty people to drink more of a fruit drink later and to give higher subjective value ratings to the pleasantness, attractiveness, and monetary value of the drink. This was all with no awareness that they either saw the subliminal stimulus or had an emotional reaction. Recently, Pessiglione and colleagues[104] have also demonstrated that subliminal perception of monetary rewards can influence motivated actions (in this case, grip strength), and the action alterations corresponded to changes in brain activity (basal forebrain) and autonomic responses. This study is exemplary for its multimeasure integration in investigating reward processing. Rewards and punishments in humans influence both actions and cognitive operations and these influences can happen in the absence of our conscious awareness.

## HOW DOES REWARD AFFECT OUR BEHAVIOUR?

The mechanisms by which reward information is integrated into the planning of actions are still little understood. This section looks at the brain areas involved in the modulation of saccade programming according to available reward information. It proposes a network of areas that may be responsible for the behavioural changes seen in the experiments described above. Again, we use the eye movement system as our benchmark since neurophysiological studies involving nonhuman primates have led to an impressive level of knowledge of the underlying processes in saccade generation[88,89] and the effect of rewards[90]. Saccade-related brain areas identified in monkeys include the frontal eye field (FEF), supplementary eye field (SEF), lateral intraparietal area (including the parietal eye field, PEF), caudate nucleus, substantia nigra pars reticulata (SNr), superior colliculus (SC), cerebellum, and brainstem saccade generators.

The basal ganglia are integral to the generation of voluntary saccades[90] via their connection to the SC, the mid-brain site of saccade generation. The mechanism behind this is the regulation of inhibitory connections from the caudate to the SNr and from the SNr to the SC. SNr neurons are spontaneously very active and inhibit SC neurons. Cortical saccade-related areas converge on both the caudate nucleus of the basal ganglia and the SC. The excitatory input to the caudate inhibits the SNr activity, thereby disinhibiting the SC neurons and thus opening the gate to the cortical excitatory information being received from FEF, SEF, and PEF. The role of the basal ganglia is to select the appropriate cortical inputs for the voluntary action[91]. The command to initiate a saccade can originate from any of these cortical areas[88]. This is illustrated by the preserved ability of subjects to make saccades despite a lesion to any one of these areas. However, such lesion studies reveal the various and specific influences that these cortical areas have on saccade programming, by the changes in saccade characteristics that are observed. For example, the FEF is involved in the preparation and triggering of voluntary saccades, the SEF in the temporal sequencing of saccades, and the PEF in visuospatial integration and reflexive saccade triggering[92,93].

Several studies have demonstrated that the basal ganglia play a key role in the reward-dependent modulation of voluntary saccades. Reward-position-selective activity has been found in caudate neurons[94] that are responsible for reward-dependent bias of saccade latency. The response of the caudate reflects both the target of an upcoming saccade and the reward expected after making the movement, and that this activity is correlated with the velocity and latency of the saccades[9]. Importantly, reward-dependent modulation of behaviour depends on normal dopamine transmission in the striatum. Injecting dopamine D1 antagonist into the caudate significantly attenuated the reward-dependent saccadic reaction times[95].

All of these studies show that this basal ganglia mechanism modifies the measurable characteristics of voluntary saccades (e.g., reaction time) depending on whether they are followed by a reward. The question that now needs answering is “where does the reward information in the basal ganglia come from?”. There are two main routes. The first is via the mesencephalic dopamine neurons described earlier that encode the reward prediction error and project directly to the basal ganglia. The effect of injecting the D1 antagonist into the caudate supports the contribution of this information. The second route is via cortical areas that have themselves been modulated by reward, and the presence of spatially selective activity in the caudate supports the contribution of this flow of information.

Therefore, the reward modulation of voluntary saccades (such as antisaccades) is effected by a network of interconnected brain areas, including the mesencephalic dopamine system, the basal ganglia, and many cortical areas. The cortical areas involved in relaying reward information will include the amygdala and OFC for reward magnitude and valence, and areas of the prefrontal and premotor cortex involved in the integration of this reward value with producing the motor output (particularly FEF, SEF, ACC, and dlPFC).

When erroneous prosaccades are made during an antisaccade task, they are also modulated by reward value[56,83]. These erroneous saccades are not voluntary in nature, and therefore the neural circuitry is different, probably excluding the basal ganglia mechanism seen in voluntary saccade generation[96]. In order to make a saccade away from a suddenly appearing visual stimulus, a reflexive prosaccade towards the stimulus must be inhibited and a voluntary antisaccade in the opposite direction must be carried out[97,98]. Single-cell recordings in monkeys have shown that this process requires the suppression of saccade neurons in the FEF[99] and the SC[100]. Without sufficient inhibition before stimulus presentation, a reflexive saccade to the stimulus may result (an erroneous prosaccade). This top-down inhibition signal may originate in the frontal lobes[92], particularly the dlPFC[93], but also the SEF and parietal cortices[101]. The lower level of activation in these areas leading to an erroneous prosaccade does not affect the modulation of the saccade parameters by reward information however. The brain areas involved in executing an erroneous prosaccade are more limited, probably including only SC, FEF, and PEF. It is surprising that erroneous prosaccades are modulated by reward given both this network and the rapidity of the saccade.

The speed with which reward information can modulate saccadic programming is also noteworthy[56,83]. Within ~200 ms, the visual stimulus has been processed and its reward value has affected the programming of the saccade. The mesencephalic dopamine neurons typically respond to rewarding stimuli in less than 100 ms. The pathways that could supply the dopaminergic neurons with such short-latency visual input are the retinal projections to the lateral geniculate-visual cortex system or the SC. However, cortical processing typically takes longer than the dopaminergic responses seen[102]. Therefore, the SC may be the more plausible relay for the very short-latency activations in the dopaminergic neurons that then contribute to the modulation of the saccadic programming of the very short latency saccades. A direct connection exists between the SC and substantia nigra, and the SC has been shown to be critical for short-latency visual activation of the mesencephalic dopamine neurons[103]. With slightly longer latency saccades (e.g., antisaccades, ~330 ms), there will obviously be more time for cortical processing of the visual information to affect any subsequent stages in the process.

To our knowledge, there is no existing fMRI work on the difference between brain activity for conscious and unconscious actions; hence, the brain mechanisms discussed here are preliminary in nature. The factor that determines whether an erroneous prosaccade is recognised as an error or not may be the level of activity in higher cortical areas, such as dlPFC, ACC, OFC, FEF, and SEF. Certainly, as we have seen above, there is a *behavioural* difference in the way that the two kinds of saccades are programmed because high punishment is found to affect unconscious prosaccades differently from conscious ones. The unconscious prosaccades were speeded, and seemed to have escaped a conscious strategy to slow down on all high valence trials[56]. This conscious strategy is presumably effected by some level of inhibition from cortical areas, and therefore the absence of the inhibition implies lower input from such areas.

In summary, the network of brain areas involved in the modulation of actions by reward is large and complex. Critical areas include the mesencephalic dopamine system, the basal ganglia, and a plethora of cortical areas, each with their own function. It seems likely that reward and punishment can have an effect on action programming with little cortical processing, although the degree of cortical input may modulate the exact behavioural outcome observed. Cortical input brings with it contextual information and the conscious recognition of actions, modifying the effect of reward on action.

## FUTURE DIRECTIONS

Several new findings have been described here: the differential effect of reward and punishment on conscious and unconscious eye movements, the labile effect of reward depending on the context in which it is presented, and the rapidity of reward processing in its effect on behaviour.

The role of consciousness cannot easily be explored using an animal model. A strong, and likely incorrect, view would be to argue that all studies of reward and behaviour in nonhuman animals are studies of unconscious action only. If we want to know more about the role of consciousness in determining the effect of rewards and punishments on human behaviour, then more exacting studies detailing both the animal's knowledge of the reward and knowledge of the action outcome are necessary.

The volume of literature concerning the brain areas involved in reward processing and its effect on behaviour is extensive. The recent explosion in human neuroimaging studies of reward and punishment is helping to piece together an integrated view of the complex system involved. The behavioural findings described here place a time constraint on any proposed network of brain areas: the processing of a visual stimulus, its translation into reward value, and the influence of that reward value on action programs, can occur within 200 ms. As with most behavioural studies of this kind, the possible visual stimuli that can appear are constrained; hence, the amount of processing that must be carried out in order to determine the stimulus is limited. In a naturalistic setting, where the number of possible visual stimuli signifying reward information is limitless, this processing stage will most likely take longer.

Further work will be needed to elucidate the more precise neural architecture and mechanisms behind the effect of rewards on our behaviour, but key to this progress will be the study of contextual effects of reward, a more precise linkage of behavioural effects to neural activations, and an assay of the animal's knowledge of both the reward and action outcome.

## ACKNOWLEDGEMENTS

This work was supported by grants from the Medical Research Council and The McDonnell Foundation. We thank Michael Posner, Avishai Henik, and two anonymous reviewers for their helpful comments.

## References

1. Berridge, K.C. and Robinson, T.E. (2003) Parsing reward. *Trends Neurosci.* **26(9),** 507–513.
2. Schultz, W. (2004) Neural coding of basic reward terms of animal learning theory, game theory, microeconomics and behavioural ecology. *Curr. Opin. Neurobiol.* **14(2),** 139–147.
3. Montague, P.R. and Berns, G.S. (2002) Neural economics and the biological substrates of valuation. *Neuron* **36(2),** 265–284.
4. O'Doherty, J. (2004) Reward representations and reward-related learning in the human brain: insights from neuroimaging. *Curr. Opin. Neurobiol.* **14(6),** 769–776.
5. McClure, S.M., York, M.K., and Montague, P.R. (2004) The neural substrates of reward processing in humans: the modern role of FMRI. *Neuroscientist* **10(3),** 260–268.
6. Roesch, M.R. and Olson, C.R. (2003) Impact of expected reward on neuronal activity in prefrontal cortex, frontal and supplementary eye fields and premotor cortex. *J. Neurophysiol.* **90(3),** 1766–1789.
7. Schultz, W. (2000) Multiple reward signals in the brain. *Nat. Rev. Neurosci.* **1(3),** 199–207.
8. Watanabe, K., Lauwereyns, J., and Hikosaka, O. (2003) Neural correlates of rewarded and unrewarded eye movements in the primate caudate nucleus. *J. Neurosci.* **23(31),** 10052–10057.
9. Kawagoe, R., Takikawa, Y., and Hikosaka, O. (2004) Reward-predicting activity of dopamine and caudate neurons--a possible mechanism of motivational control of saccadic eye movement. *J. Neurophysiol.* **91(2),** 1013–1024.
10. Robbins, T.W. and Everitt, B.J. (1996) Neurobehavioural mechanisms of reward and motivation. *Curr. Opin. Neurobiol.* **6(2),** 228–36.
11. Berridge, K.C. and Robinson, T.E. (1998) What is the role of dopamine in reward: hedonic impact, reward learning, or incentive salience? *Brain Res. Brain Res. Rev*. **28(3)**, 309–369.
12. Schultz, W. (1998) Predictive reward signal of dopamine neurons. *J. Neurophysiol.* **80(1),** 1–27.
13. Schultz, W. (2002) Getting formal with dopamine and reward. *Neuron* **36(2),** 241–263.
14. Louilot, A., Le Moal, M., and Simon, H. (1986) Differential reactivity of dopaminergic neurons in the nucleus accumbens in response to different behavioral situations. An in vivo voltammetric study in free moving rats. *Brain Res*. **397(2)**, 395–400.
15. Schultz, W. (1986) Responses of midbrain dopamine neurons to behavioral trigger stimuli in the monkey. *J. Neurophysiol*. **56(5),** 1439–1461.
16. Ljungberg, T., Apicella, P., and Schultz, W. (1992) Responses of monkey dopamine neurons during learning of behavioral reactions. *J. Neurophysiol*. **67(1),** 145–163.
17. Schultz, W. and Dickinson, A. (2000) Neuronal coding of prediction errors. *Annu. Rev. Neurosci.* **23**, 473–500.
18. Hollerman, J.R. and Schultz, W. (1998) Dopamine neurons report an error in the temporal prediction of reward during learning. *Nat. Neurosci*. **1(4),** 304–309.
19. McClure, S.M., Berns, G.S., and Montague, P.R. (2003) Temporal prediction errors in a passive learning task activate human striatum. *Neuron* **38(2)**, 339–346.
20. O'Doherty, J. et al. (2003) Temporal difference models and reward-related learning in the human brain. *Neuron* **38(2),** 329–337.
21. Seymour, B. et al. (2004) Temporal difference models describe higher-order learning in humans. *Nature* **429(6992)**, 664–667.
22. Jensen, J. et al. (2007) Separate brain regions code for salience vs. valence during reward prediction in humans. *Hum. Brain Mapp*. **28(4),** 294–302.
23. Berns, G.S. et al. (2001) Predictability modulates human brain response to reward. *J. Neurosci.* **21(8),** 2793–2798.
24. Knutson, B. et al. (2001) Anticipation of increasing monetary reward selectively recruits nucleus accumbens. *J. Neurosci*. **21(16),** RC159.
25. Hikosaka, O., Sakamoto, M.,and Usui, S. (1989) Functional properties of monkey caudate neurons. III. Activities related to expectation of target and reward. *J. Neurophysiol.* **61(4)**, 814–832.
26. Hollerman, J.R., Tremblay, L., and Schultz, W. (1998) Influence of reward expectation on behavior-related neuronal activity in primate striatum. *J. Neurophysiol.* **80(2),** 947–963.
27. Delgado, M.R. et al. (2000) Tracking the hemodynamic responses to reward and punishment in the striatum. *J. Neurophysiol.* **84(6),** 3072–3077.
28. Amador, N., Schlag-Rey, M., and Schlag, J. (2000) Reward-predicting and reward-detecting neuronal activity in the primate supplementary eye field. *J. Neurophysiol.* **84(4)**, 2166–2170.
29. Campos, M. et al. (2005) Supplementary motor area encodes reward expectancy in eye-movement tasks. *J. Neurophysiol.* **94(2)**, 1325–1335.
30. Baxter, M.G. and Murray, E.A. (2002) The amygdala and reward. *Nat. Rev. Neurosci.* **3(7)**, 563–573.
31. Nishijo, H., Ono, T., and Nishino, H. (1988) Single neuron responses in amygdala of alert monkey during complex sensory stimulation with affective significance. *J. Neurosci.* **8(10),** 3570–3583.
32. Ono, T., Nishijo, H., and Nishino, H. (2000) Functional role of the limbic system and basal ganglia in motivated behaviors. *J. Neurol.* **247(Suppl 5)**, V23–32.
33. Hamann, S. and Mao, H. (2002) Positive and negative emotional verbal stimuli elicit activity in the left amygdala. *Neuroreport* **13(1),** 15–19.

34. Hommer, D.W. et al. (2003) Amygdalar recruitment during anticipation of monetary rewards: an event-related fMRI study. *Ann. N. Y. Acad. Sci.* **985**, 476–478.
35. Anderson, A.K. et al. (2003) Dissociated neural representations of intensity and valence in human olfaction. *Nat. Neurosci.* **6(2)**, 196–202.
36. Small, D.M. et al. (2003) Dissociation of neural representation of intensity and affective valuation in human gustation. *Neuron* **39(4)**, 701–711.
37. Pratt, W.E. and Mizumori, S.J. (1998) Characteristics of basolateral amygdala neuronal firing on a spatial memory task involving differential reward. *Behav. Neurosci*. **112(3),** 554–570.
38. Kringelbach, M.L. (2005) The human orbitofrontal cortex: linking reward to hedonic experience. *Nat. Rev. Neurosci*. **6(9),** 691–702.
39. Thorpe, S.J., Rolls, E.T., and Maddison, S. (1983) The orbitofrontal cortex: neuronal activity in the behaving monkey. *Exp. Brain Res*. **49(1),** 93–115.
40. Rolls, E.T. (2000) The orbitofrontal cortex and reward. *Cereb. Cortex* **10(3)**, 284–294.
41. Elliott, R. et al. (2003) Differential response patterns in the striatum and orbitofrontal cortex to financial reward in humans: a parametric functional magnetic resonance imaging study. *J. Neurosci.* **23(1),** 303–307.
42. O'Doherty, J. et al. (2000) Sensory-specific satiety-related olfactory activation of the human orbitofrontal cortex. *Neuroreport* **11(2),** 399–403.
43. Damasio, A.R. (1994) *Descartes' Error: Emotion, Reason and the Human Brain*. Grosset/Putnam, New York.
44. Rolls, E.T. et al. (1994) Emotion-related learning in patients with social and emotional changes associated with frontal lobe damage. *J. Neurol. Neurosurg. Psychiatry* **57(12),** 1518–1524.
45. Tremblay, L. and Schultz, W. (1999) Relative reward preference in primate orbitofrontal cortex. *Nature* **398(6729),** 704–708.
46. Schall, J.D. (2001) Neural basis of deciding, choosing and acting. *Nat. Rev. Neurosci.* **2(1),** 33–42.
47. O'Doherty, J. et al. (2003) Dissociating valence of outcome from behavioral control in human orbital and ventral prefrontal cortices. *J. Neurosci*. **23(21),** 7931–7939.
48. O'Doherty, J. et al. (2001) Abstract reward and punishment representations in the human orbitofrontal cortex. *Nat. Neurosci*. **4(1),** 95–102.
49. Breiter, H.C. et al. (2001) Functional imaging of neural responses to expectancy and experience of monetary gains and losses. *Neuron* **30(2),** 619–639.
50. Small, D.M. et al. (2001) Changes in brain activity related to eating chocolate: from pleasure to aversion. *Brain* **124(Pt 9),** 1720–1733.
51. Rolls, E.T. et al. (2003) Representations of pleasant and painful touch in the human orbitofrontal and cingulate cortices. *Cereb. Cortex* **13(3),** 308–317.
52. Nitschke, J.B. et al. (2006) Functional neuroanatomy of aversion and its anticipation. *Neuroimage* **29(1),** 106–116.
53. Sarinopoulos, I. et al. (2006) Brain mechanisms of expectation associated with insula and amygdala response to aversive taste: implications for placebo. *Brain Behav. Immun.* **20(2),** 120–132.
54. Seymour, B., Singer, T., and Dolan, R. (2007) The neurobiology of punishment. *Nat. Rev. Neurosci.* **8(4),** 300–311.
55. Elliott, R., Dolan, R.J., and Frith, C.D. (2000) Dissociable functions in the medial and lateral orbitofrontal cortex: evidence from human neuroimaging studies. *Cereb. Cortex* **10(3),** 308–317.
56. Blaukopf, C.L. and DiGirolamo, G.J. (2006) Differential effects of reward and punishment on conscious and unconscious eye movements. *Exp. Brain Res*. **174(4),** 786–792.
57. Shima, K. and Tanji, J. (1998) Role for cingulate motor area cells in voluntary movement selection based on reward. *Science* **282(5392),** 1335–1338.
58. Posner, M.I. and DiGirolamo, G.J. (1998) Executive attention: conflict, target detection and cognitive control. In *The Attentive Brain*. Parasuraman, R., Ed. MIT Press, Cambridge, MA. pp. 401–423.
59. Amiez, C., Joseph, J.P., and Procyk, E. (2006) Reward encoding in the monkey anterior cingulate cortex. *Cereb. Cortex* **16(7),** 1040–1055.
60. Knutson, B. et al. (2003) A region of mesial prefrontal cortex tracks monetarily rewarding outcomes: characterization with rapid event-related fMRI. *Neuroimage* **18(2),** 263–272.
61. Ullsperger, M. and von Cramon, D.Y. (2003) Error monitoring using external feedback: specific roles of the habenular complex, the reward system, and the cingulate motor area revealed by functional magnetic resonance imaging. *J. Neurosci*. **23(10),** 4308–4314.
62. Cohen, M.X., Heller, A.S., and Ranganath, C. (2005) Functional connectivity with anterior cingulate and orbitofrontal cortices during decision-making. *Brain Res. Cogn. Brain Res.* **23(1),** 61–70.
63. Gehring, W.J. and Willoughby, A.R. (2002) The medial frontal cortex and the rapid processing of monetary gains and losses. *Science* **295(5563),** 2279–2282.
64. Taylor, S.E. (1991) Asymmetrical effects of positive and negative events: the mobilization-minimization hypothesis. *Psychol. Bull.* **110(1),** 67–85.
65. Williams, Z.M. et al. (2004) Human anterior cingulate neurons and the integration of monetary reward with motor responses. *Nat. Neurosci.* **7(12),** 1370–1375.
66. Ito, S. et al. (2003) Performance monitoring by the anterior cingulate cortex during saccade countermanding. *Science* **302(5642)**, 120–122.

67. Blair, K. et al. (2006) Choosing the lesser of two evils, the better of two goods: specifying the roles of ventromedial prefrontal cortex and dorsal anterior cingulate in object choice. *J. Neurosci*. **26(44),** 11379–11386.
68. Goldman-Rakic, P.S. (1987) Circuitry of primate prefrontal cortex and regulation of behavior by representational memory. In *Handbook of Physiology. Section 1. The Nervous System*. Plum, F., Ed. American Physiological Society, Bethesda, MD. pp. 373–417.
69. Kobayashi, S. et al. (2002) Influence of reward expectation on visuospatial processing in macaque lateral prefrontal cortex. *J. Neurophysiol.* **87(3),** 1488–1498.
70. Roesch, M.R. and Olson, C.R. (2004) Neuronal activity related to reward value and motivation in primate frontal cortex. *Science* **304(5668),** 307–310.
71. Holroyd, C., Larsen, J., and Cohen, J. (2004) Context dependence of the event-related brain potential associated with reward and punishment. *Psychophysiology* **41(2),** 245.
72. Nieuwenhuis, S. et al. (2005) Activity in human reward-sensitive brain areas is strongly context dependent. *Neuroimage* **25(4),** 1302–1309.
73. Akitsuki, Y. et al. (2003) Context-dependent cortical activation in response to financial reward and penalty: an event-related fMRI study. *Neuroimage* **19(4),** 1674–1685.
74. Watanabe, M. et al. (2002) Coding and monitoring of motivational context in the primate prefrontal cortex. *J. Neurosci.* **22(6),** 2391–2400.
75. Nakahara, H. et al. (2004) Dopamine neurons can represent context-dependent prediction error. *Neuron* **41(2),** 269–280.
76. Walton, M.E., Devlin, J.T., and Rushworth, M.F. (2004) Interactions between decision making and performance monitoring within prefrontal cortex. *Nat. Neurosci.* **7(11)**, 1259–1265.
77. Dickinson, A. and Balleine, B.W. (1994) Motivational control of goal-directed action. *Anim. Learn. Behav.* **22(1),** 1–18.
78. Hayhoe, M. and Ballard, D. (2005) Eye movements in natural behavior. *Trends Cogn. Sci.* **9(4),** 188–194.
79. Kawagoe, R., Takikawa, Y., and Hikosaka, O. (1998) Expectation of reward modulates cognitive signals in the basal ganglia. *Nat. Neurosci.* **1(5)**, 411–416.
80. Takikawa, Y. et al. (2002) Modulation of saccadic eye movements by predicted reward outcome. *Exp. Brain Res.* **142(2),** 284–291.
81. Watanabe, M. et al. (2001) Behavioral reactions reflecting differential reward expectations in monkeys. *Exp. Brain Res.* **140(4),** 511–518.
82. Jazbec, S. et al. (2005) Cognitive control under contingencies in anxious and depressed adolescents: an antisaccade task. *Biol. Psychiatry* **58(8),** 632–639.
83. Blaukopf, C.L. and DiGirolamo, G.J. (2005) The automatic extraction and use of information from cues and go signals in an antisaccade task. *Exp. Brain Res.* **167(4),** 654–659.
84. Fischer, B., Gezeck, S., and Hartnegg, K. (2000) On the production and correction of involuntary prosaccades in a gap antisaccade task. *Vision Res.* **40(16),** 2211–2217.
85. Trommershauser, J., Maloney, L.T., and Landy, M.S. (2003) Statistical decision theory and trade-offs in the control of motor response. *Spat. Vis*. **16(3–4),** 255–275.
86. Della Libera, C. and Chelazzi, L. (2006) Visual selective attention and the effects of monetary rewards. *Psychol. Sci.* **17(3),** 222–227.
87. Berridge, K.C. and Winkielman, P. (2003) What is an unconscious emotion: the case for unconscious 'liking'. *Cogn. Emotion* **17**, 181–211.
88. Wurtz, R.H. and Goldberg, M.E., Eds. (1989) *The Neurobiology of Saccadic Eye Movements.* Elsevier, Amsterdam.
89. Sparks, D.L. (2002) The brainstem control of saccadic eye movements. *Nat. Rev. Neurosci.* **3(12),** 952–964.
90. Hikosaka, O., Nakamura, K., and Nakahara, H. (2006) Basal ganglia orient eyes to reward. *J. Neurophysiol.* **95(2),** 567–584.
91. Hikosaka, O., Takikawa, Y., and Kawagoe, R. (2000) Role of the basal ganglia in the control of purposive saccadic eye movements. *Physiol. Rev.* **80(3),** 953–978.
92. Gaymard, B. et al. (1998) Cortical control of saccades. *Exp. Brain Res.* **123(1–2),** 159–163.
93. Pierrot-Deseilligny, C. et al. (2002) Effects of cortical lesions on saccadic: eye movements in humans. *Ann. N. Y. Acad. Sci.* **956**, 216–229.
94. Lauwereyns, J. et al. (2002) A neural correlate of response bias in monkey caudate nucleus. *Nature* **418(6896),** 413–417.
95. Nakamura, K. and Hikosaka, O. (2006) Role of dopamine in the primate caudate nucleus in reward modulation of saccades. *J. Neurosci.* **26(20),** 5360–5369.
96. Munoz, D.P. and Everling, S. (2004) Look away: the anti-saccade task and the voluntary control of eye movement. *Nat. Rev. Neurosci.* **5(3),** 218–228.
97. Everling, S. and Fischer, B. (1998) The antisaccade: a review of basic research and clinical studies. *Neuropsychologia* **36(9),** 885–899.
98. Curtis, C.E. and D'Esposito, M. (2003) Success and failure suppressing reflexive behavior. *J. Cogn. Neurosci.* **15(3),** 409–418.
99. Everling, S. and Munoz, D.P. (2000) Neuronal correlates for preparatory set associated with pro-saccades and antisaccades in the primate frontal eye field. *J. Neurosci.* **20(1)**, 387–400.
100. Everling, S., Dorris, M.C., and Munoz, D.P. (1998) Reflex suppression in the anti-saccade task is dependent on prestimulus neural processes. *J. Neurophysiol.* **80(3)**, 1584–1589.
101. Ford, K.A. et al. (2005) Neural processes associated with antisaccade task performance investigated with event-related FMRI. *J. Neurophysiol.* **94(1),** 429–440.
102. Thorpe, S.J. and Fabre-Thorpe, M. (2001) Neuroscience. Seeking categories in the brain. *Science* **291(5502),** 260–263.
103. Comoli, E. et al. (2003) A direct projection from superior colliculus to substantia nigra for detecting salient visual events. *Nat. Neurosci.* **6(9)**, 974–980.
104. Pessiglione, M., Schmidt, L., Draganski, B., Kalisch, R., Lau, H., Dolan, R. J., et al. (in press). How the brain translates money into force: A neuroimaging study of subliminal motivation. *Science.*
